# 1-Deoxynojirimycin containing *Morus alba* leaf-based food modulates the gut microbiome and expression of genes related to obesity

**DOI:** 10.1186/s12917-024-03961-9

**Published:** 2024-04-03

**Authors:** Varun Jaiswal, Mi-Jin Lee, Ju Lan Chun, Miey Park, Hae-Jeung Lee

**Affiliations:** 1 Department of Food and Nutrition, College of BioNano Technology, Gachon University, Seongnam, Gyeonggi-do 13120 Republic of Korea; 2 Institute for Aging and Clinical Nutrition Research, Gachon University, Seongnam, Gyeonggi-do 13120 Republic of Korea; 3 Department of Companion Animal Industry, College of Health Sciences, Wonkwang University, Iksan, Jeollabuk-do 54538 Republic of Korea; 4 Animal Welfare Research Team, Rural Development Administration, National Institute of Animal Science, Wanju, Jeollabuk-do 55365 Republic of Korea; 5 Department of Health Sciences and Technology, GAIHST, Gachon University, Incheon, 21999 Republic of Korea

**Keywords:** Obesity, Microbiome, Transcriptome, Natural products, Diabetes, Pathways

## Abstract

**Background:**

Obesity is a serious disease with an alarmingly high incidence that can lead to other complications in both humans and dogs. Similar to humans, obesity can cause metabolic diseases such as diabetes in dogs. Natural products may be the preferred intervention for metabolic diseases such as obesity. The compound 1-deoxynojirimycin, present in *Morus* leaves and other sources has antiobesity effects. The possible antiobesity effect of 1-deoxynojirimycin containing *Morus alba* leaf-based food was studied in healthy companion dogs (*n* = 46) visiting the veterinary clinic without a history of diseases. Body weight, body condition score (BCS), blood-related parameters, and other vital parameters of the dogs were studied. Whole-transcriptome of blood and gut microbiome analysis was also carried out to investigate the possible mechanisms of action and role of changes in the gut microbiome due to treatment.

**Results:**

After 90 days of treatment, a significant antiobesity effect of the treatment food was observed through the reduction of weight, BCS, and blood-related parameters. A whole-transcriptome study revealed differentially expressed target genes important in obesity and diabetes-related pathways such as MLXIPL, CREB3L1, EGR1, ACTA2, SERPINE1, NOTCH3, and CXCL8. Gut microbiome analysis also revealed a significant difference in alpha and beta-diversity parameters in the treatment group. Similarly, the microbiota known for their health-promoting effects such as *Lactobacillus ruminis*, and *Weissella hellenica* were abundant (increased) in the treatment group. The predicted functional pathways related to obesity were also differentially abundant between groups.

**Conclusions:**

1-Deoxynojirimycin-containing treatment food have been shown to significantly improve obesity. The identified genes, pathways, and gut microbiome-related results may be pursued in further studies to develop 1-deoxynojirimycin-based products as candidates against obesity.

**Supplementary Information:**

The online version contains supplementary material available at 10.1186/s12917-024-03961-9.

## Introduction

In humans, the incidence of obesity has been increasing globally, almost tripling since 1975, and World Health Organization (WHO) has recognized it as an epidemic. Worldwide, 650 million adults are estimated to be obese, and at least 2.8 million people die from obesity every year. Obesity and diabetes are linked, and obesity can contribute to the pathogenesis of diabetes and the development of its complications [1, 2]. The incidence of obesity in dogs is on the rise, as in humans [3] and can enhance the threat of other diseases, such as metabolic and cardiovascular diseases [4, 5]. Obesity is a multifactorial disorder associated with several pathways, which makes it challenging to treat [6–9].

Herbal dietary supplements have high availability in the general population and are less toxic than general drug therapies against diseases, especially during long-term use. The leaves of *Morus alba* (MA) have been used as a functional food in different countries because of their high nutritional value and the presence of several phytocompounds, such as alkaloids, flavonoids, glycosides, and phenolic acids, which are considered responsible for its health-promoting activities [10–13]. MA leaf extract has potential activity against various metabolic diseases, particularly obesity and diabetes [11, 14]. The compound 1-deoxynojirimycin which is primarily found in these leaves, is considered responsible for both antiobesity and antidiabetes effects in human and animal models [15–18]. In our previous study, the effect of similar diets on aged dogs also highlighted the anti-obesity potential; however, no significant difference was observed between the control and treatment groups, which might be due to the small number of animals considered in the study [19].

Obesity and diabetes are complex diseases, and multiple mechanisms have been proposed to explain the pathophysiology of these diseases that are associated with several genes and pathways. Therefore, whole-transcriptome analysis of the studied animals may be utilized to explore changes in the expression of almost all possible genes to obtain mechanistic insights and possible targets for treatment [20–23]. Differentially expressed genes in the treatment group associated with important pathways in obesity and diabetes were important candidates for further analysis. These genes can explain the improvement in obesity parameters in the treatment group and open avenues for establishing their role in disease through further research. Similarly, gut microbiota is strongly associated with obesity and diabetes [24–26]. The gut microbiota regulates obesity by influencing central appetite, fatty acid tissue composition, chronic inflammation, circadian rhythm, energy production, and absorption from food [25, 27]. Diet is the key influencer in the shaping of the gut microbiome, which can influence the pathology of obesity and metabolic diseases. Gut microbiome analysis was also conducted on the dogs before and after the treatment diet. In similar studies, we observed changes in diversity and other parameters related to the microbiome; however, the results were not significant because of the small number of subjects [19, 28]. Therefore, a relatively large number of animals was included in the current study to achieve significant results.

## Results

### Body weight (BW), body condition score (BCS), and blood parameters

**Fig. 1:**
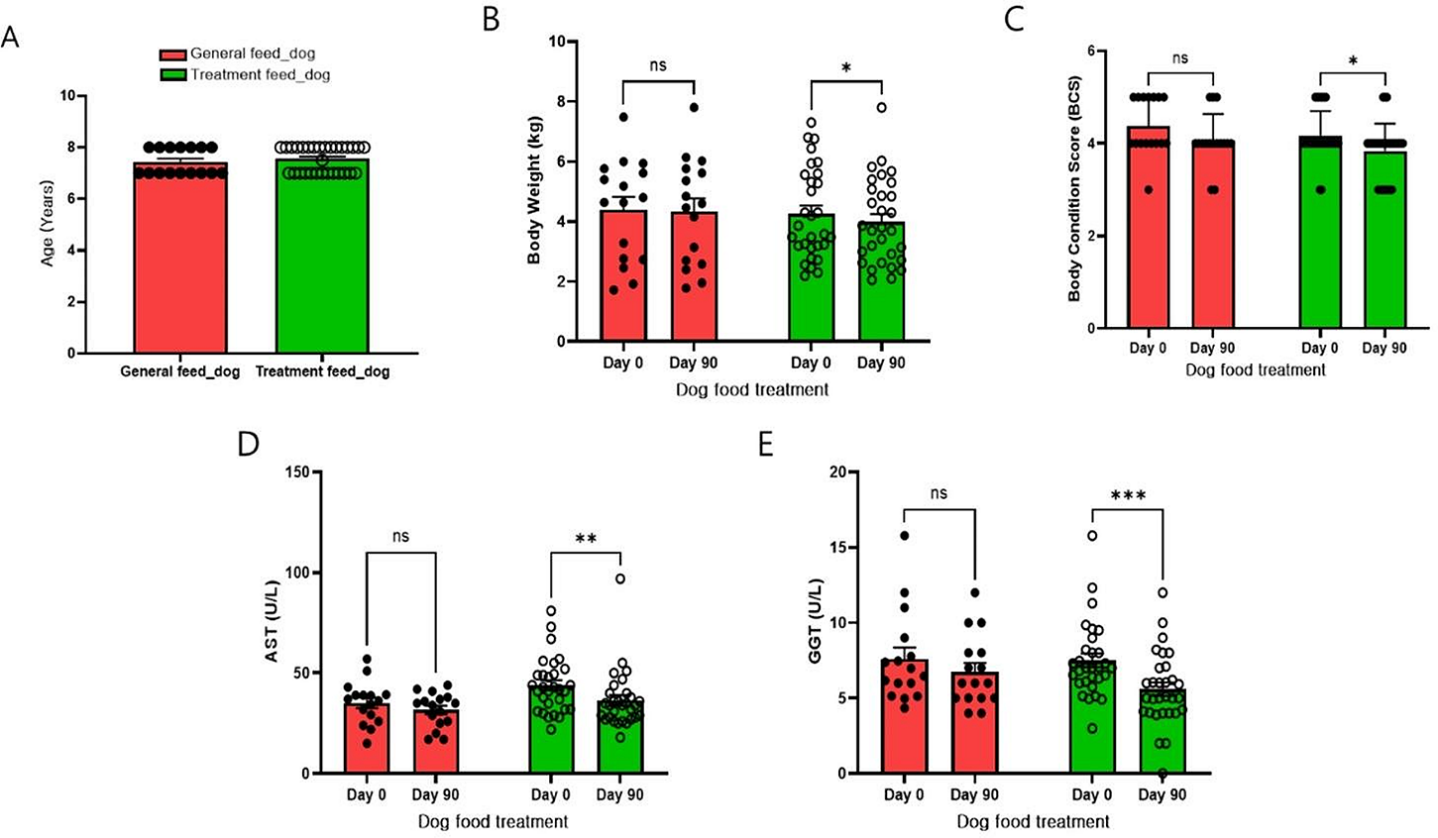
Result of the comparison between the general or treatment feed dog group for 90 days. **(A)** Age, **(B)** body weight, **(C)** body condition score, **(D)** aspartate aminotransferase (AST), and **(E)** gamma-glutamyltransferase (GGT). All data are presented as mean ± standard error of the mean (SEM). * *p* < 0.05, ** *p* < 0.01, and *** *p* < 0.001. Non-significant comparisons between the groups are not mentioned in the figure

The mean age of the dogs is 7.44 ± 0.51 (years) in the general feed group and 7.55 ± 0.50 (years) in the treatment feed group (Fig. 1A). Body weight (BW) and body condition score (BCS) were not significantly different between the control and treatment feeding dog groups (Fig. 1B and C) on day 0 (before treatment). However, 90 days after the treatment feed administration, BW and BCS were significantly lower in the treatment feed group than in the general feed group. Importantly, the difference between the groups was not significant after 90 days of the treatment. In the blood chemistry test, aspartate aminotransferase (AST) (Fig. 1D) and gamma-glutamyltransferase (GGT) levels (Fig. 1E) among the parameters were significantly reduced at 90 days after treatment in the treatment feed group. In all animals, the values of GGT and AST were in the reference range i.e. 0–8 and 18–56 U/L for GGT and AST respectively [29, 30]. In both the general and treatment feed groups, there were no significant differences in weight, heart rate, respiratory rate, or body temperature prior to the test. The levels were within the normal range throughout the test period, and no specific findings were observed (Tables S1 and S2).

### Preprocessing and alignment of reads

High-quality RNA-sequencing data were generated using next-generation sequencing to study the whole-transcriptome in both groups. A high percentage of good-quality reads was obtained in the quality control experiment (Table S3). Similarly, a high alignment rate (> 96%) of these reads was obtained, which justifies the high quality of the assembled transcriptomic data utilized in the current study (Table S3).

### Assembly and expression analysis

**Fig. 2:**
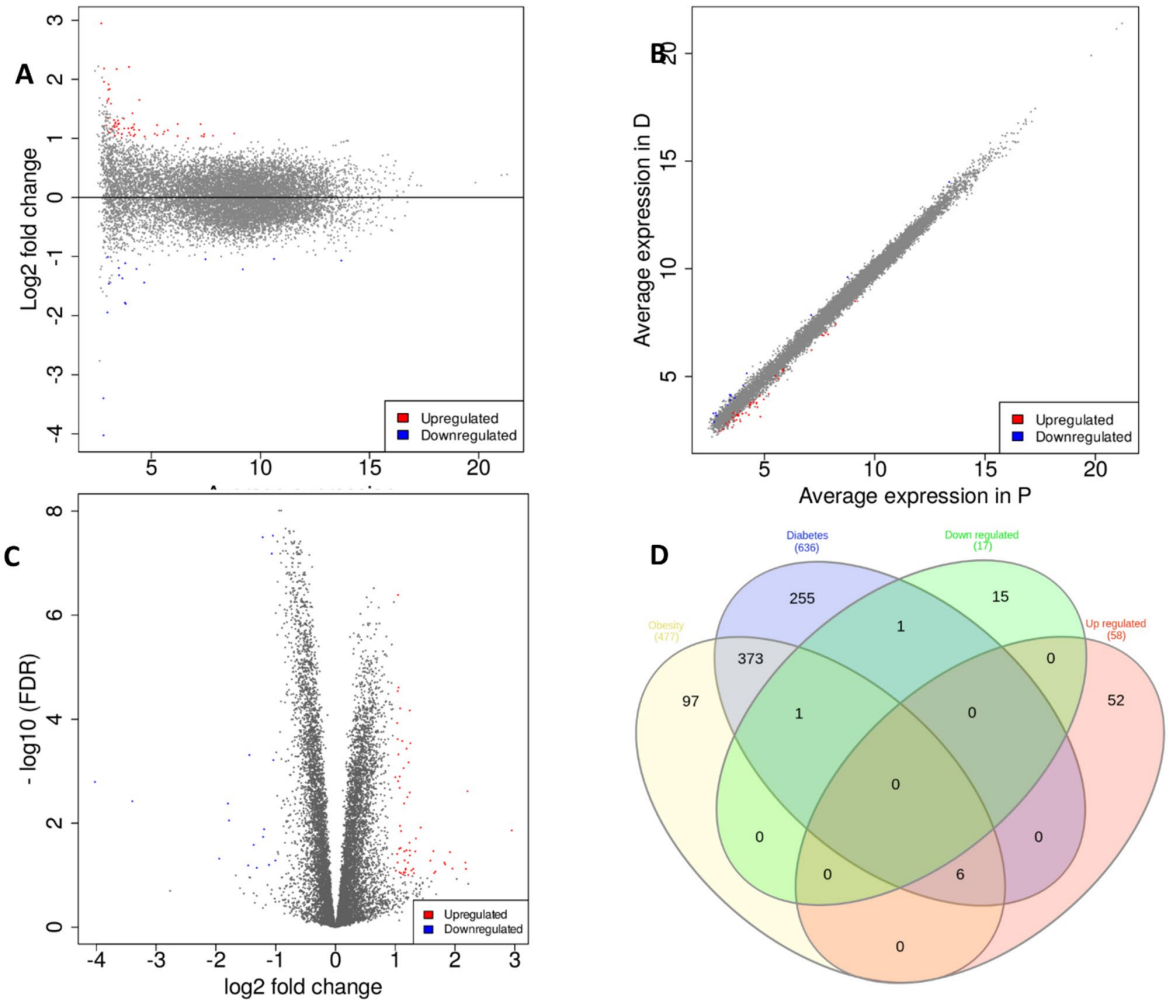
Plots showing differential gene expression through **(A)** MA **(B)** scattered, **(C)** volcano plots, and **(D)** Venn diagram differentially expressed genes (DEGs) with genes associated with obesity and diabetes pathways in the Kyoto Encyclopedia of Genes and Genomes (KEGG)

The assembly of all genes/transcripts from all samples was used for the expression studies. The expression of all genes/transcripts assembled in all the samples was obtained as read counts and fragments per kilobase of transcripts per million mapped reads (FPKM). Differentially expressed genes (DEGs) were identified by comparing groups according to the selected thresholds. A total of 17 and 60 DEGs were found to be down-regulated and up-regulated, respectively, in the treatment groups compared with untreated dogs. The DEGs were visualized using scattered, MA and volcano plots (Fig. 2).

### Functional enrichment analysis

Functional enrichment of the significantly up-regulated DEGs revealed that most genes were associated with cellular processes, followed by biological regulation and metabolic processes in the biological process category. Most genes were consistently associated with cellular and anatomical entities in the cellular components category. Most DEGs were enriched in the binding categories of the molecular functions. Gene-specific transcriptional regulators and transmembrane signal receptors were the most enriched categories of protein classes. Pathway enrichment analysis revealed that 30 diverse pathways were associated with the upregulated DEGs (Figure S1).

Most down-regulated DEGs were associated with cellular processes in the biological process category, followed by metabolic processes. Similarly, the most enriched category in the cellular component category was the cellular anatomical entity. Most DEGs were associated with molecular function in the binding categories. Metabolite interconversion enzymes are the most enriched categories in the protein classes. Pathway enrichment analysis revealed that five diverse pathways, including interleukin mediated by cytokine and chemokine signaling pathways were associated with downregulated genes (Figure S2).

### DEGs associated with obesity and diabetes pathways

**Fig. 3:**
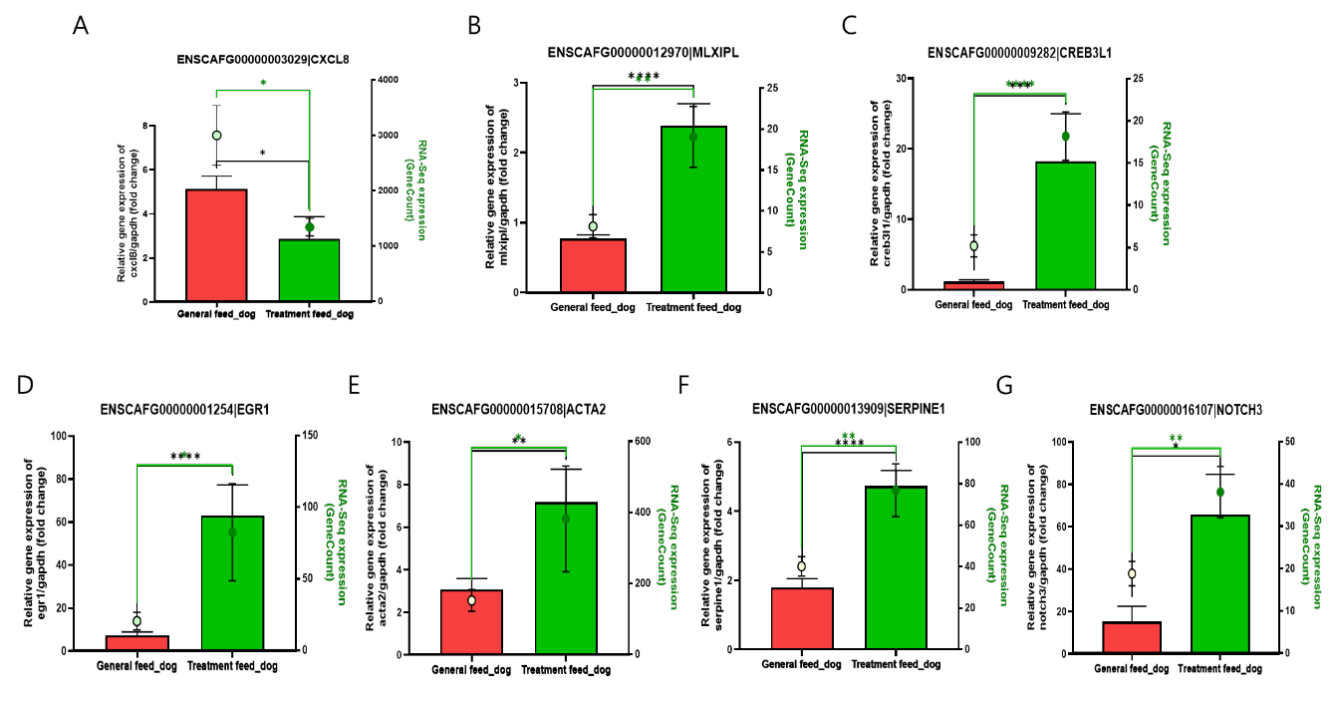
RNA-Seq and reverse transcription polymerase chain reaction (RT-PCR) expressions of obesity and diabetes-related genes between the general and treatment feed dog groups. **(A)** CXCL8, **(B)** MLXIPL, **(C)** CREB3L1, **(D)** EGR1, **(E)** ACTA2, **(F)** SERPINE1, and **(G)** NOTCH3. The red and green lines present RT-PCR and RNA-Seq (gene counts values) respectively, between the general and treatment feed dog groups. All data are presented as mean ± standard error of the mean (SEM). * *p* < 0.05, ** *p* < 0.01, *** *p* < 0.001, and **** *p* < 0.0001

DEGs common in both obesity and diabetes pathways genes reported in the Kyoto Encyclopedia of Genes and Genomes (KEGG) database were identified from the subset analysis (Fig. 2). Six genes (MLXIPL, CREB3L1, EGR1, ACTA2, SERPINE1, and NOTCH3) were upregulated and one gene (CXCL8) was downregulated in the treatment group. The expression of all these genes was validated by real-time polymerase chain reaction (PCR) analysis which showed expression results similar to those obtained in the RNA-seq experiment (Fig. 3).

### Gut microbiome and diversity analysis

A total of 8,296,559 paired-end reads were used for the gut microbiome analysis. After filtering and pre-processing, 3,332,394 feature reads/amplicon sequence variants (ASVs)/operational taxonomic units (OTUs) were obtained for different analyses comprising 16,500 unique features/ASVs/OTUs identified from all samples.

**Fig. 4:**
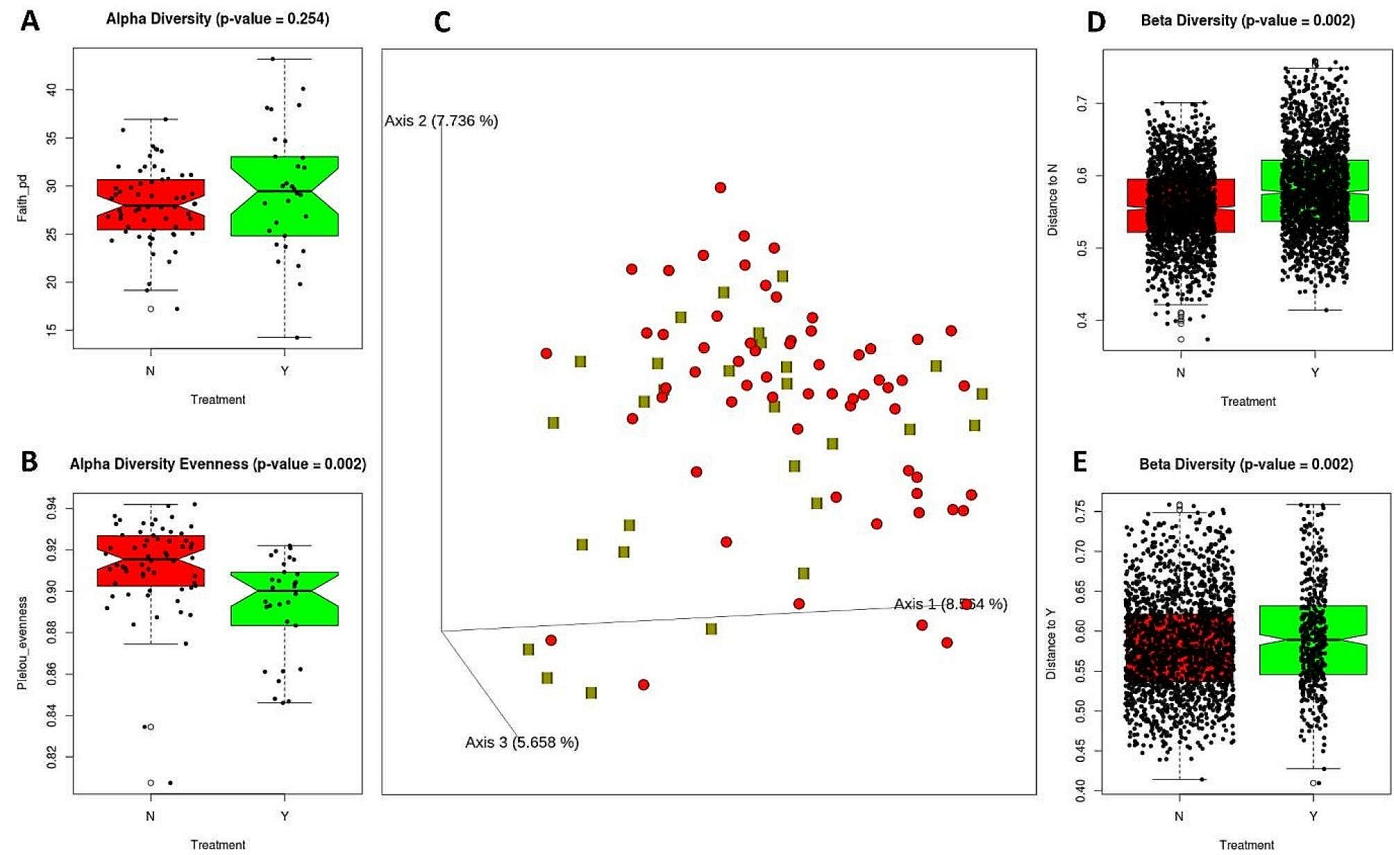
Plots showing alpha and beta diversities between animals with and without treatment. **(A)** Faith phylogeny diversity, **(B)** Pielou evenness, **(C)** 3-D plot depicting beta-diversity through unweighted UniFrac distance, **(D)** beta-diversity distance to without treatment (N), and **(E)** distance to treatment. Red color is used for the without treatment group (N) and green is used for the treatment group (Y)

An increase in alpha-diversity was observed in the treatment groups; however, this difference was not significant. The treatment groups showed small but significant differences in the Pielou evenness of alpha diversity (Fig. 4). A slight increase in the beta-diversity distance was also observed in the treatment group, which was again significant in the permutational multivariate analysis of variance according to the p-value cutoff (Fig. 3).

### Taxonomy analysis of the samples

**Fig. 5:**
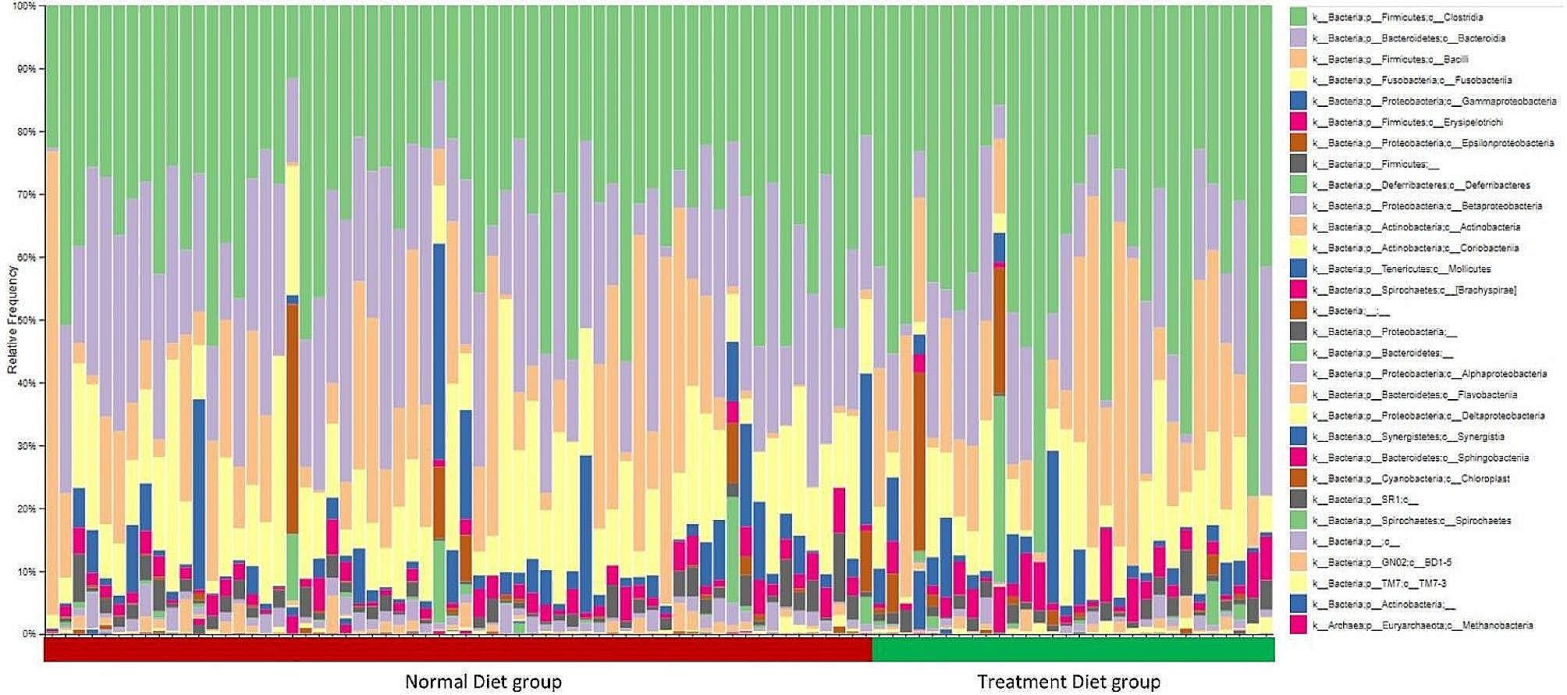
Bar plot depicting taxonomic distribution in all samples

Taxonomic annotations of all samples were visualized using bar plots to study the relative frequencies of the different taxa present in all samples (Fig. 5). The predominant phyla present in the samples were Firmicutes and Bacteroidetres; Clostridia and Bacteroidia were among the most abundant classes in most of the samples (Fig. 5).

### Differential abundance of taxa between the groups

**Fig. 6:**
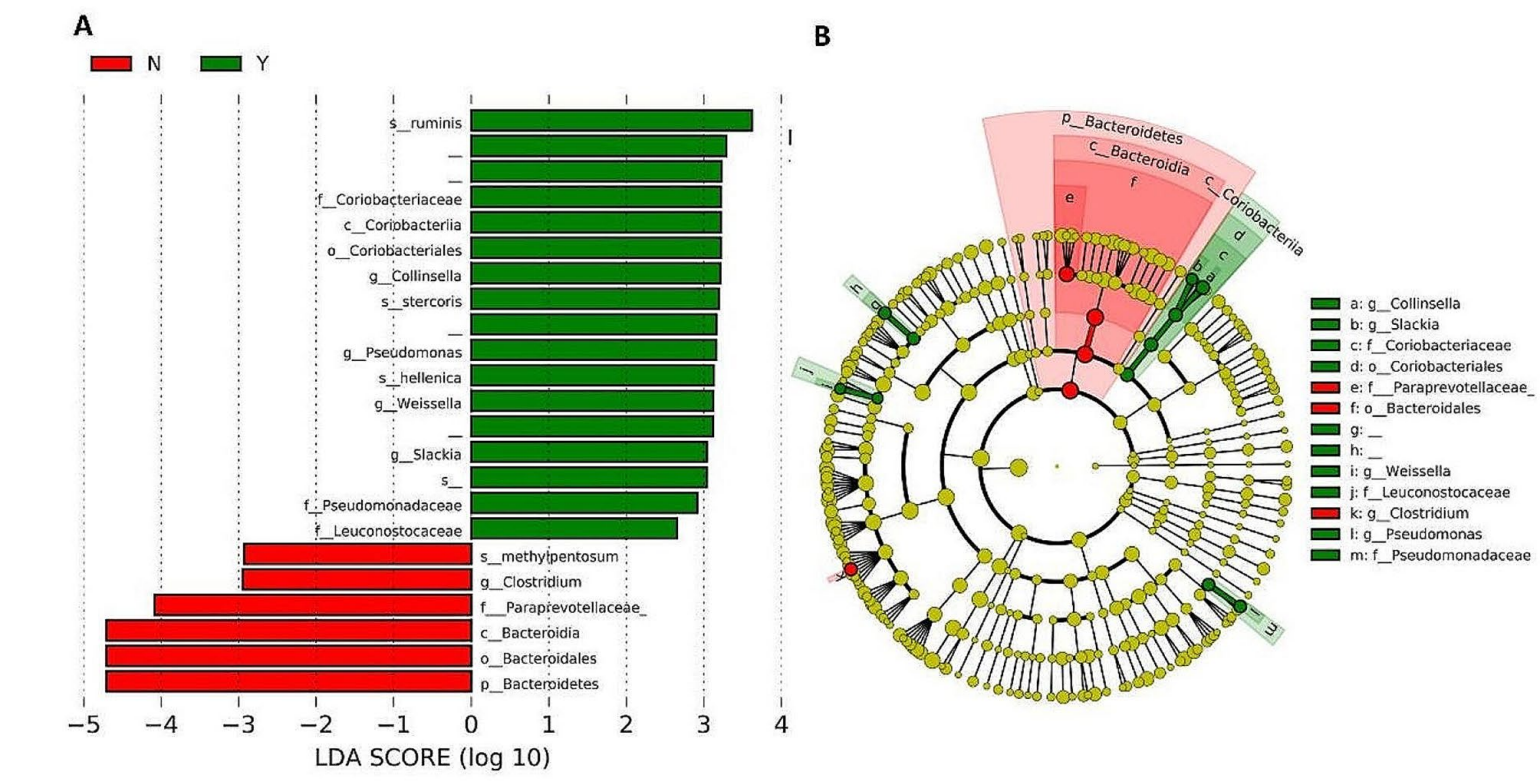
Differentially abundant taxa in the treatment and nontreatment groups. **(A)** Bar plot depicting abundant taxa and **(B)** cladogram. N is the general feed dog group and Y is the treatment feed dog group. s_: species; g_: genus; f_: family; c_: class; o_: order; p_: phylum

The classes Coriobacteriia and Bacteroidia were found in the treatment and non-treatment groups, respectively (Fig. 6). *Lactobacillus ruminis*, *Weissella hellenica*, and *Collinsella stercoris* were abundant in the treatment group, whereas *Clostridium methylpentosum* was abundant in the untreated group (Fig. 5). Species abundant in treatment, such as *Lactobacillus ruminis, are* used as probiotics, and are associated with health benefits such as immune enhancement [31]. Similarly, *Weissella hellenica* has been found to be probiotic [32].

### Correlation of gut microbiome with obesity-related factors

**Fig. 7:**
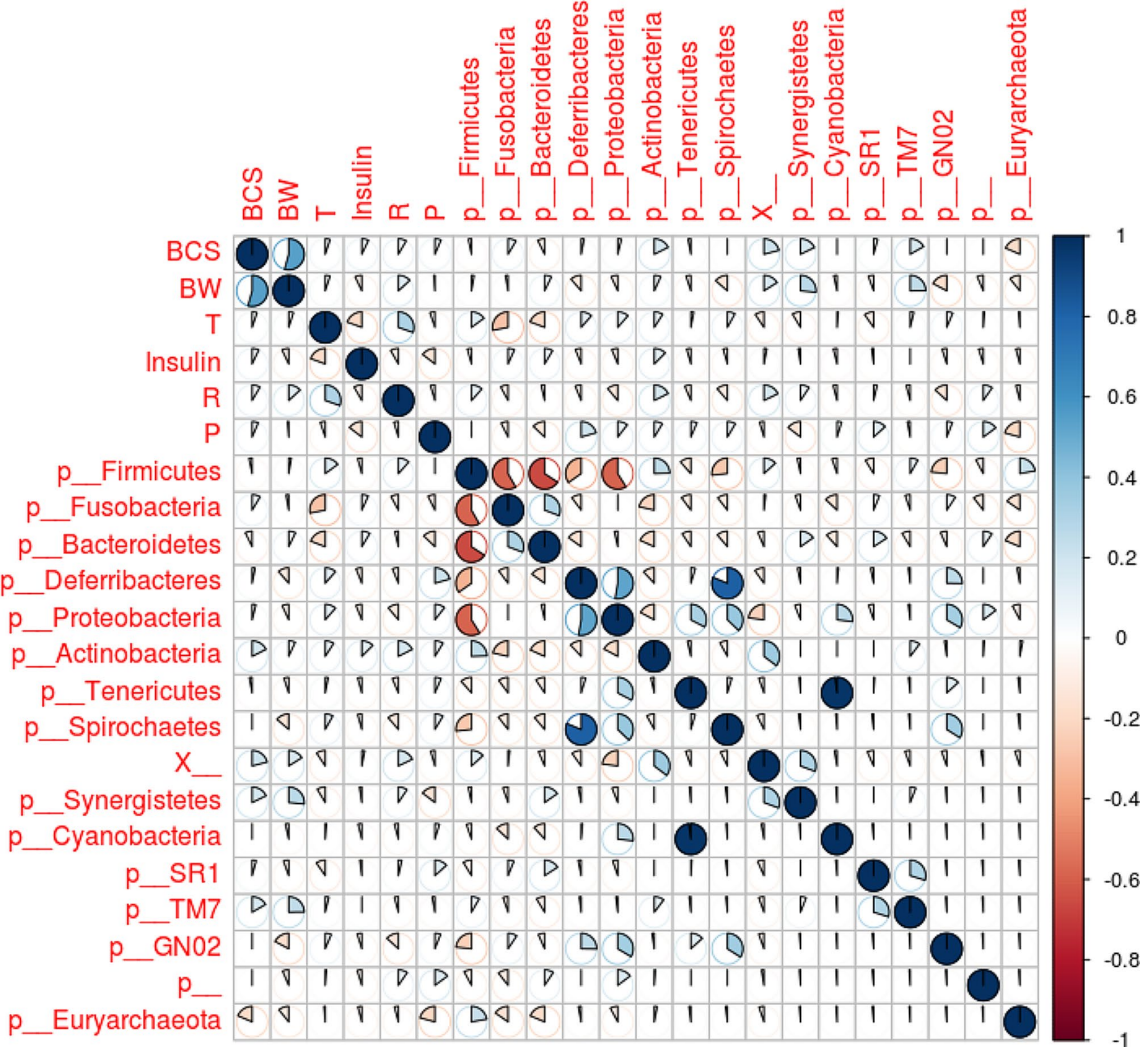
Visualization of correlation matrix among obesity-related factors and gut microbiome. BCS: body condition score; BW: body weight; T: body temperature; P: pulse rate; R: respiratory rate; X_: taxonomy unassigned; p_: phylum unassigned; Color bar on the right represent correlation values (-1 to 1); the circles are filled clockwise for positive values, and anti-clockwise for negative values

No strong correlation between gut microbiome and obesity related-factors was observed at the phylum or species level as the correlation coefficient was between − 0.29 and 0.41 (Fig. 7 and Figure S3). An obvious correlation was observed between body weight and body condition score (BCS) in the correlation analysis, as BCS can be considered a measure of obesity associated with body weight (Fig. 7).

### Functional potential of the gut microbiome

**Fig. 8:**
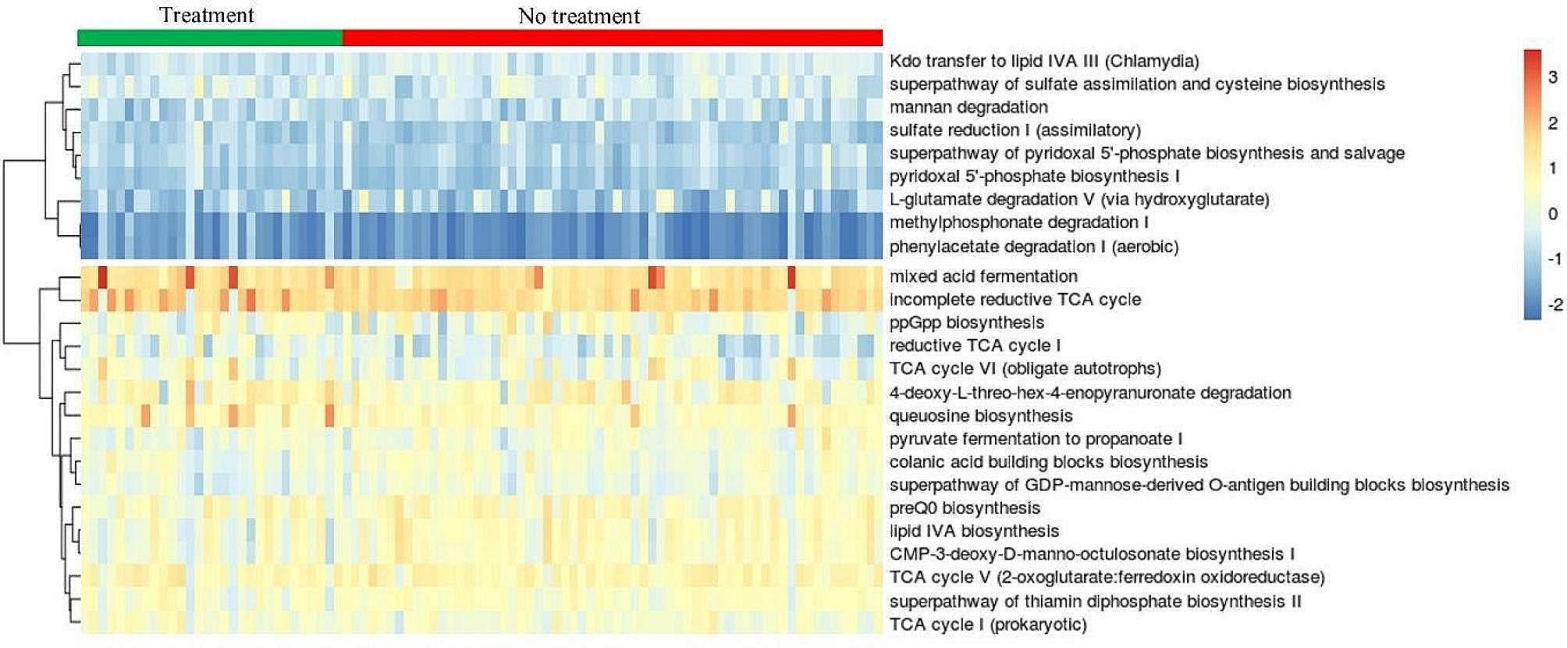
Heatmap showing the abundance of pathways, which were differentially abundant in the treatment group

Prediction of the functional potential of gut microbiome through ASV was performed in terms of enzyme commission (EC) numbers, KEGG orthologs (KOs), and pathway abundances. Pathway abundances are the main high-level prediction outputs that were calculated through the structured mappings of EC gene families to pathways. A total of 25 different pathways were found to be differentially abundant among the studied groups according to both the expected Benjamini-Hochberg corrected p values (< 0.05) of the Wilcoxon test and Welch’s t-test (Fig. 8).

## Discussion

In earlier studies, 1-deoxynojirimycin was found in the leaves of different mulberry species, silkworms, and the metabolites of some bacteria (such as species of *Streptomyces* and *Bacillus*) known for their different biological properties, especially antiobesity and antidiabetes effects [28, 33]. In the current study, the antiobesity effects of the 1-deoxynojirimycin containing MA based diet on the reduction of weight and BCS score and improvement in blood-related parameters, such as AST and GGT, were investigated to explore the possible molecular mechanism and role of the gut microbiome in the treatment group. Among liver enzymes, especially GGT and oxidative stress are positively associated with obesity [34]. GGT is a known marker of liver dysfunction and reduction of GGT may have positive effect in number of liver associated diseases [35–37]. Furthermore, information regarding lipid metabolites in the blood might be useful to explore the role of lipid metabolism in the anti-obesity effect of the treatment diet and should be pursued in future studies. RNA-seq-based whole-transcriptome studies have proven vital in deciphering the role of treatments in different diseases and conditions, including obesity [38–40]. In all samples, the high percentage of good quality-reads (mean value > 98.6%) and alignment rate (mean value > 95%) of these reads on the reference genome indicated the high quality of the transcriptome generated and utilized in the current study (Table S3). DEGs may provide information regarding target genes and pathways altered by the provided treatments or conditions. Improvement in obesity in the treatment feed group, which may also improve metabolic diseases such as diabetes, inspired the study of the association of DEGs in the treatment group in pathways related to both diabetes and obesity. Venn diagram analysis revealed that seven DEGs (CXCL8, MLXIPL, CREB3L1, EGR1, NOTCH3, ACTA2, and SERPINE1) were associated with both obesity and diabetes-associated pathways, including non-alcoholic fatty liver disease, insulin resistance, thermogenesis, and apelin signaling pathways. These DEGs are important target genes for both obesity and diabetes (TGBOD). The expression of all these genes was again cross-checked using RT-PCR, which was found to follow the same pattern as that observed in the RNA-Seq (whole transcriptome) analysis. Among the TGBOD, CXCL8 was found to be down-regulated in the treatment group, which is consistent with previous studies showing that the expression of CXCL8 has a positive relationship with obesity [41, 42].

The gut microbiome is highly associated with diet and affects metabolic diseases, including diabetes and obesity [26, 27, 43]. Therefore, changes in the microbiome have been studied to explore their role in the improving obesity. A slight increase in alpha-diversity and beta diversity distance was observed in the treatment group, but it was only significant in the latter case. Similarly, a slight but significant change in the evenness of the alpha diversity was observed. A significant increase in beta diversity distance may be associated with improved obesity, as in different studies, a significant change in beta diversity among obese and non-obese subjects was observed [44]. Further, species such as *Lactobacillus ruminis* and *Weissella hellenica* were found to be abundant in the treatment groups. These species are used as probiotics and are responsible for several health benefits, including weight control [45]. Probiotic strains such as *Lactobacillus* have also been found to improve glycemia in preclinical studies [46]. Additionally, the functional potential of the gut microbiota may provide a functional basis for the anti-obesity effects of MA. The functional abundance of the gut microbiota predicted through pathway abundance was used to highlight the functions that were differentially abundant in the treatment group. Twenty-five significantly differential abundant pathways were shown in the results of both tests. Among them, various pathways such as the tricarboxylic acid (TCA) cycle [47], lipid isobutyryl coenzyme A (IVA) biosynthesis [48], mannan degradation [49], cysteine biosynthesis [50], L-glutamate degradation V [51], and super pathway of thiamine diphosphate biosynthesis II [52] (Fig. 7) may be linked with obesity/diabetes as these pathways or their related compounds are known to be associated with obesity in the literature [47, 51, 53–56]. Therefore, it would be interesting to study the effects of these pathways on the gut microbiome and their roles in obesity. Previous studieshave highlighted the potential of 1-deoxynojirimycin against obesity and diabetes in animal models and humans, and the current study not only supports this but also provides insight into the possible target genes and pathways, as well as the putative role of the gut microbiome in its antiobesity activity. The current study strengthens the basis for the development of 1-deoxynojirimycin and 1-deoxynojirimycin containing potential foods (such as MA, silkworms and bacterial broth) as supplements and/or treatments for obesity in companion animals and humans.

## Conclusion

1-Deoxynojirimycin containing *Morus alba* leaf-based food has shown significant improvement in obesity and related parameters. These antiobesity effects may be due to changes in the gut microbiome and expression of genes linked to both obesity and diabetes-associated pathways. The identified genes, pathways, and gut microbiome-related results may be pursued in further research to develop the 1-deoxynojirimycin-based supplement/treatment against obesity in animals and humans.

## Materials and methods

### Animals

#### Treatment feed developed for participant dogs visiting animal hospitals

Dogs with more than six out of the nine stages of the physical BCS by veterinary tests and dogs with a history of disease or suffering from any disease were excluded. Informed consent was obtained from the guardians of all the test participants before inclusion in the study and the testing method was approved by the Animal Testing Ethics Committee (PTB-2021-IACUC-012-A). All animals (*n* = 46) that met the inclusion and exclusion criteria visiting the animal hospital were considered in the study without statistical calculation of the sample size, as a larger number of animals were incorporated in the study than in several previous similar studies [19, 28, 57, 58]. Weight and BCS were measured by a veterinarian in the hospital before feeding (day 0) and at 30, 60, and 90 days after feeding. The treatment feed was provided once at a dose of 100 g/day per 5 kg of body weight for 90 days. For the control group, veterinarian-trained guardians provides the same amount of existing general feed (treatment feed without the component of *Morus alba* leaves) without snacks for 90 days.

Fecal and blood samples were collected from all animals before and after treatment withgeneral or treatment feeds for 90 days. After collecting blood, the blood was left unattended for more than 30 min. After centrifugation at 4 °C for 10 min at 400 × g, the serum samples were stored at -80 °C until use. Whole blood samples before and after 90 days of feed intake were collected from animals with RNAprotect® Animal Blood Tubes (QIAGEN, Germany) for RNA-Seq. Insulin (ELISA, Biovendor, Heidelberg, Germany), serum AST (AST assay kits, Asanpharm, Seoul, Korea), and GGT (GGT assay kits, Asanpharm, Seoul, Korea) were analyzed using commercial kits according to the manufacturer’s protocol. The animal fecal samples of 0 and 90 days were stored at -80 °C until use.

### Animal signalment

The participants were privately owned dogs visiting animal hospitals. All test subjects were included in the study after informed consent was obtained from their guardians. The recruited information on dogs, classified by breed, is presented in Table S4. All 46 dogs, 16 for general feed and 30 for treatment feed, were recruited at seven years or older from small-to large-size breeds. animals from each group were randomly selected through Experimental Animal Allotment Program [59]. Information about the breeds, sex and neutered and unneutered statuses of all animals considered in the study is provided in the table (Table S5). At the end of the study, all the animals were unharmed and made free with their guardians.

### Information on treatment food and animal maintenance

The nutritional composition of the treatment food developed by Erebon Co. (Erebon, Icheon, Republic of Korea) is shown in Table 1. It was designed for elderly dogs or dogs at high risk of metabolic diseases and was used in this study. The main ingredients of the formulated treatment feed were *Morus alba* leaves along with hydrolyzed chicken, oats, hydrolyzed dried chicken, chicken fat, powdered cellulose, oats, bit pulp, inulin, omega 3, vitamins C and E, and a mineral premix (calcium, potassium, magnesium, carbohydrate, and salt). As shown in previous mouse experiments, the amount *Morus alba* leaves used in this study was determined based on the amount of 1-deoxynojirimycin [60]. Unlike previous mouse experiments, this pet food used hydrolyzed *Morus alba* leaf powder. In addition, values obtained from the analysis institution (Korea Institute for Health Promotion, Korea) were converted and applied to the amount of pet food in this experiment. Furthermore, the feed contains all the appropriate nutrients for dogs and is prepared as an extrusion with a standard structure. The chicken components in the feed may be responsible for its palatability. All dogs were fed individually according to the their weight (100 g/day per 5 kg) for 90 days under the direction of the veterinarian.

**Table 1:** Information on the nutrition of treatment food

Nutrition	Pet food per 100 g
Protein	27.10 g
Fat	12.20 g
Carbohydrate (NFE)	41.20 g
Dietary Fiber	12.76 g
Calcium	1.10 g
Phosphorus	0.90 g
Sodium	0.32 g
Magnesium	0.10 g
Omega 6	2.10 g
Omega 3	0.77 g
L-carnitine	30.00 mg
Taurine	0.30 g
1-deoxynojirimycin (from *Morus alba* leaf)	0.02 mg
Metabolic energy	343.00 kcal

### RNA sequencing

Total RNA was extracted from the dog’s blood before and after treatments with general or treatment feed for 90 days, using the RNeasy Protect Animal Blood Kit (QIAGEN, Hilden, Germany). A total of 500 ng of RNA was used to prepare whole transcriptome sequencing libraries. The next-generation sequencing (NGS) and RNA‑Seq analysis were performed as previously described [28].

### RNA-Seq analysis for assembly and expression analysis

Raw paired-end reads obtained from the NGS platform were analyzed in a quality control study (QCS) by fastpV-0.23.2 [61]. Good-quality reads after (filtering, error removal, and trimming) were used for alignment-based assembly in subsequent steps. The reference genome of *Canis lupus familiaris* (dog), CanFam 3.1 was used to align good-quality reads using HISAT2 [62]. For each sample, the alignment results were stored in sequence alignment map files, which were further transformed into BAM files as per the prerequisite of the next assembly analysis. Reads were assembled by aligning BAM files using StringTie [63]. For the differential expression analysis, the assembly files of all samples were combined using Python program (prepDE.py) provided with StringTie. Finally, a file with read count values of the assembled genes/transcripts for all samples was prepared for further differential expression studies. The study design information and gene count files were used in the expression analysis using iDEP.6 [64]. DESeq2 and EdgeR methods were utilized to study differential expression with default cut-off values (false discovery rate (FDR) < 0.1 and fold change (gene expression) of 2) for the selection of DEGs.

### Functional enrichment of DEGs

Functional enrichment of both up and down-regulated DEGs was conducted using Protein ANalysis THrough Evolutionary-Relationships (PANTHER). The DEGs were enriched according to pathways, molecular functions, biological processes, cellular components, and protein classes [65]. All DEGs identified through Ensembl IDs were submitted as query to the PANTHER classification system for selecting *Canis*. *lupus familiaris* as the mark organism. The output results were saved in the CSV and image file formats. Further, KEGG pathways were used to identify the DEGs associated with both obesity and diabetes. Subset analysis was performed on DEGs and genes from obesity and diabetes pathways in the KEGG database.

### DEGs common in obesity and diabetes pathways

Genes associated with obesity and diabetes pathways present in the KEGG pathway database were collected. Subset analysis was performed using InteractiVenn [66] to identify common upregulated and down-regulated DEGs associated with obesity and diabetes.

### Real-time PCR analysis

The expression of all the DEGs associated with both obesity and diabetes pathways was studied and validated using real-time PCR analysis. Dog blood RNA was reverse-transcribed to cDNA (iScript cDNA synthesis kit; BioRad, Hercules, CA, USA). Real-time PCR was performed using theTB Green Master Mix (TaKaRa Bio, Otsu, Japan) and analyzed using the QuantStudio3 PCR system (Thermo Fisher Scientific, San Jose, CA, USA). The sequences of primer (5’- 3’) utilized for RT-PCR are presented in Table S6, and the normalization of expression was carried out according to the internal glyceraldehyde 3-phosphate dehydrogenase (GAPDH) gene of the dog.

### Gut microbiome sequence analysis

Pair-end reads of amplicon sequences of the V3–V4 region from the 16 S ribosomal sequences were analyzed using Quantitative Insights into Microbial Ecology (QIIME-2) [67]. Quality control analysis was conducted on the reads from all samples incorporated into QIIME-2 prior to the QCS. After the visualization of both forward and reverse reads through the “qiime tools view module” the Divisive amplicon denoising algorithm 2 (DADA2) module was utilized to denoise, trim, filter chimeras, and remove low quality reads [68]. Amplicon sequence variants (ASVs) obtained after DADA2 analysis were further analyzed. Multiple sequence alignments were performed using MAFFT to align the ASVs for phylogenetic studies [69]. A phylogenetic tree was constructed from this alignment using FastTree program [70].

### Taxonomic annotation

The q2-feature-classifier module of QIIME-2 was used for taxonomical annotation of ASVs using the Greengenes 13_8 99% OTUs-based taxonomy classifier [71]. This classifier uses the naïve Bayes algorithm for taxonomic annotation. Finally, the taxonomic annotation presented all samples as a bar plot drawn using the qiime taxa barplot” module [72].

### Alpha and beta-diversity analysis

Alpha diversity was analyzed based on both the richness and evenness of the microbiome community. Community richness was studied using Shannon’s diversity index, observed features, and Faith’s phylogenetic diversity. Community evenness was studied using Pielou’s evenness measure. Likewise, beta-diversity was studied through community dissimilarity using both qualitative and quantitative measures, specifically by the Jaccard and Bray Curtis distance measures. Additionally, beta-diversity was studied by considering the phylogenetic relationship between features through qualitative and quantitative measures of community dissimilarity using unweighted UniFrac and weighted UniFrac distances, respectively.

### Abundance of taxa in the groups

The differential abundance of taxa in both groups was analyzed using linear discriminant analysis effect size (LEfSe) [73]. The ASV table, metadata information, and 7th level (species level) collapsed taxonomy results were used for differential abundance analysis using LEfSe. In the LEfSe analysis, high cut-off parameters such as *p*-values of 0.001 and the one-against-all strategy were considered. Cladograms and bar graphs were constructed to visualize the differences in the microbiome communities among the groups [73].

### Correlation analysis of obesity-related factors with the gut microbiome

Pearson correlation through the R program was utilized to calculate the correlation between microbiome and different experimental parameters (such as body weight, BCS, etc.) with 2nd-level (phylum level) and 7th-level (species level) collapsed taxonomy results utilized for the calculation of Pearson-correlation through the R program. Finally, the correlation matrix was plotted using the Corrplot function in R.

### Functional potential of gut microbiome

The functional potential of the gut microbiome via ASV was predicted using PICRUSt2 [74]. The ASV table and sequence information from the qiime-2 pipeline were used for the functional prediction of EC, KOs, and pathway abundance in the samples from both groups. Pathway abundances were the main high-level prediction outputs that were calculated through the structured mapping of EC gene families to pathways. Differentially abundant functions of the microbial community in the groups were identified through pathways that were differentially expressed by ALDeX2 [75].

### Statistical analysis

Data were analyzed using GraphPad Prism 9.2. The difference between treatment and control animal groups was studied through two-way analysis of variance (ANOVA) and values of *p* < 0.05 were considered significant.

### Electronic supplementary material

Below is the link to the electronic supplementary material.

Supplementary Material 1: Supplimentary tables (Table S1-S6) and figures (Figures S1-S3).

## Data Availability

The high-throughput sequencing datasets generated and utilized in this study were submitted to the publicly accessible NCBI repository (BioProject: PRJNA1028815).

## Abbreviations

AST: aspartate aminotransferase
ASVs: amplicon sequence variants
BCS: body condition score
BW: body weight
DADA2: divisive amplicon denoising algorithm 2
DEGs: differentially expressed genes
EC: enzyme Commission numbers
FDR: false discovery rate
PANTHER: Protein ANalysis THrough Evolutionary-Relationships
FPKM: fragments per kilobase of transcript per million mapped reads
GGT: gamma-glutamyltransferase
KEGG: kyoto encyclopedia of genes and genomes
KOs: KEGG orthologs
LEfSe: linear discriminant analysis effect size
MA: Morus alba
NGS: next-generation sequencing
OTUs: operational taxonomic units
WHO: world health organization
