# Supplementary material for: 1-Deoxynojirimycin containing Morus alba leaf-based food modulates the gut microbiome and expression of genes related to obesity
Source: BMC Vet Res. 2024 Apr 3;20:133. doi: 10.1186/s12917-024-03961-9 (PMC10988916; doi:10.1186/s12917-024-03961-9)
Supplement: Supplementary file 1 — Supplementary Material 1: Supplimentary tables (Table S1-S6) and figures (Figures S1-S3). [file 12917_2024_3961_MOESM1_ESM.pdf]

Table S1. Physical examination results through the clinical efficacy evaluation of this study.

Items (Unit)		Day 0	Day 90
Body weight (kg)	General Feed	4.33 ± 1.65	4.34 ± 1.75
	Diabetes Feed	4.24 ± 1.52	4.00 ± 1.41
Body temperature (°C)	General Feed	38.59 ± 0.59	38.87 ± 0.37
	Diabetes Feed	38.97 ± 0.40	38.86 ± 0.27
Heart rate (per minute)	General Feed	109.25 ± 18.81	112.00 ± 16.59
	Diabetes Feed	112.17 ± 19.62	115.20 ± 11.65
Respiration rate (per minute)	General Feed	22.94 ± 3.17	27.88 ± 6.39
	Diabetes Feed	27.50 ± 4.92	24.47 ± 2.01

Mean ± standard deviation.

Table S2. Results of complete blood chemistry, and electrolyte test through the clinical efficacy evaluation of this study.

Items (Unit)		Day 0	Day 90	Reference
WBC (10^9/L)	General Feed	11.22 ± 3.70	13.70 ± 9.10	5.05 - 16.76
	Diabetes Feed	10.92 ± 4.19	11.09 ± 4.68	
RBC (10^{12}/L)	General Feed	6.90 ± 0.95	6.85 ± 0.70	5.65 - 8.87
	Diabetes Feed	7.07 ± 0.82	6.64 ± 0.75	
HGB (g/dL)	General Feed	15.45 ± 2.54	14.89 ± 1.64	13.1 - 20.5
	Diabetes Feed	15.96 ± 1.70	15.13 ± 1.68	
HCT (%)	General Feed	48.08 ± 6.63	46.29 ± 4.42	37.3-61.7
	Diabetes Feed	49.22 ± 4.14	47.09 ± 4.43	
MCV (fL)	General Feed	69.71 ± 3.24	67.71 ± 2.69	61.6 - 73.5
	Diabetes Feed	69.89 ± 3.93	71.11 ± 2.88	
MCH (pg)	General Feed	22.33 ± 1.49	21.76 ± 1.41	21.2 - 25.9
	Diabetes Feed	22.63 ± 1.52	22.81 ± 1.23	
MCHC (g/dL)	General Feed	32.05 ± 1.62	32.13 ± 1.36	32.0 - 37.9
	Diabetes Feed	32.38 ± 1.27	32.08 ± 1.02	
PLT (10^3/μL)	General Feed	388.00 ± 249.04	465.94 ± 219.93	148 - 484
	Diabetes Feed	385.83 ± 179.98	398.37 ± 134.87	
ALT (U/dL)	General Feed	85.97 ± 88.86	83.81 ± 96.04	19 - 70
	Diabetes Feed	101.43 ± 135.61	59.43 ± 35.45	
ALP (U/dL)	General Feed	68.73 ± 62.34	100.94 ± 127.21	15.0 - 127.0
	Diabetes Feed	46.07 ± 25.95	87.43 ± 175.69	
CRE (mg/dL)	General Feed	0.60 ± 0.23	0.51 ± 0.16	0.5-1.3
	Diabetes Feed	0.58 ± 0.13	0.57 ± 0.14	
Na (mmol/L)	General Feed	147.16 ± 3.79	144.94 ± 3.20	144-154
	Diabetes Feed	146.89 ± 2.94	146.47 ± 1.92	
K (mmol/L)	General Feed	5.17 ± 0.51	5.02 ± 0.35	4.1 - 5.3

	Diabetes Feed	5.05 ± 0.33	5.00 ± 0.34	
Cl (mmol/L)	General Feed	110.61 ± 3.66	110.44 ± 5.44	105.0 – 116.0
	Diabetes Feed	111.37 ± 3.56	113.22 ± 2.99	

Mean ± standard deviation.

WBC, white blood cells; RBC, red blood cells; HGB, hemoglobin; HCT, hematocrit; MCV, mean cell volume; MCH, mean cell hemoglobin; MCHC, mean cell hemoglobin concentration; PLT, platelets; ALT, alanine aminotransferase; ALP, alkaline phosphatase; CRE, creatinine; Na, Natrium; K, potassium; Cl, chloride.

Table S3: Preprocessing and alignment result of all 92 samples.

**Sr. No.**	**ID**	**# Good Reads in millions (in percentage)**	**Alignment rate**
1	D1	69.781086 M (98.618843%)	96.45%
2	D2	66.353858 M (98.774808%)	94.47%
3	D3	65.589644 M (98.893148%)	96.47%
4	D4	59.640530 M (97.881373%)	96.55%
5	D5	69.180148 M (98.227722%)	96.74%
6	D6	78.576742 M (98.961710%)	96.92%
7	D7	74.838002 M (98.854102%)	95.80%
8	D8	72.902930 M (98.695830%)	96.32%
9	D9	71.521724 M (98.800531%)	94.88%
10	D10	62.779276 M (98.827727%)	95.96%
11	D11	61.397626 M (98.576361%)	95.46%
12	D12	62.825068 M (99.067228%)	96.27%
13	D13	61.306896 M (98.275546%)	96.17%
14	D14	71.810060 M (98.892188%)	95.45%
15	D15	75.684756 M (98.833122%)	96.82%
16	D16	79.740206 M (98.694620%)	95.99%
17	D17	62.952828 M (98.459016%)	95.56%
18	D18	66.755614 M (98.498060%)	96.37%
19	D19	74.599424 M (98.090869%)	96.18%
20	D20	70.673122 M (98.424755%)	96.81%
21	D21	69.098392 M (98.668298%)	96.74%
22	D22	69.645330 M (98.557045%)	96.63%
23	D23	58.711434 M (98.566071%)	96.33%

24	D24	64.396544 M (98.967501%)	95.60%
25	D25	67.849432 M (98.677952%)	93.74%
26	D26	76.096134 M (97.899623%)	94.08%
27	D27	72.372828 M (98.079407%)	96.46%
28	D28	80.180828 M (97.752518%)	95.60%
29	D29	78.647420 M (97.851776%)	96.33%
30	D30	72.406960 M (98.152748%)	93.37%
31	D31	68.607088 M (98.759047%)	97.02%
32	D32	66.095392 M (99.045551%)	95.78%
33	D33	74.330492 M (98.065084%)	95.95%
34	D34	72.147180 M (98.338493%)	96.20%
35	D35	77.611056 M (98.333148%)	95.93%
36	D36	62.626198 M (99.056657%)	96.91%
37	D37	71.280028 M (98.042957%)	94.77%
38	D38	71.406136 M (98.720463%)	94.40%
39	D39	75.588820 M (99.091766%)	96.92%
40	D40	68.025370 M (98.358508%)	96.13%
41	D41	78.129754 M (99.095791%)	96.15%
42	D42	69.873112 M (98.736258%)	95.80%
43	D43	62.109174 M (98.671045%)	96.07%
44	D44	77.385118 M (98.387923%)	95.95%
45	D45	77.520640 M (98.701978%)	94.51%
46	D46	71.216646 M (98.607730%)	96.41%
47	P1	63.209442 M (98.792600%)	97.24%
48	P2	75.594856 M (98.998476%)	94.87%
49	P3	61.675730 M (98.771111%)	96.34%
50	P4	59.999352 M (98.589355%)	96.00%
51	P5	77.085150 M (98.745405%)	96.64%
52	P6	63.588004 M (98.177794%)	96.43%
53	P7	70.331042 M (98.444000%)	96.69%
54	P8	62.226362 M (98.722313%)	96.66%
55	P9	70.530906 M (98.730999%)	95.79%
56	P10	69.131190 M (98.087426%)	96.37%
57	P11	68.002542 M (98.925126%)	96.54%

58	P12	61.361180 M (98.290206%)	96.14%
59	P13	87.703574 M (98.720795%)	95.33%
60	P14	67.624516 M (98.851346%)	96.00%
61	P15	72.254322 M (99.016068%)	96.11%
62	P16	65.064330 M (98.703328%)	96.22%
63	P17	61.137512 M (98.731751%)	95.47%
64	P18	63.631086 M (98.472783%)	96.39%
65	P19	72.074152 M (97.732254%)	94.95%
66	P20	58.851344 M (99.053574%)	97.18%
67	P21	58.782920 M (97.986881%)	95.96%
68	P22	71.392840 M (98.913382%)	95.56%
69	P23	59.409720 M (98.981995%)	95.94%
70	P24	68.570684 M (98.914662%)	96.93%
71	P25	60.343120 M (98.891470%)	95.70%
72	P26	64.946324 M (98.347362%)	95.70%
73	P27	80.796966 M (98.993357%)	96.99%
74	P28	63.549372 M (98.986622%)	95.98%
75	P29	61.042510 M (98.847928%)	95.13%
76	P30	65.679444 M (98.923198%)	95.95%
77	P31	76.777436 M (98.461937%)	96.22%
78	P32	77.272116 M (98.760359%)	96.29%
79	P33	63.902696 M (98.816372%)	95.17%
80	P34	75.025490 M (98.777360%)	95.40%
81	P35	67.690286 M (98.696549%)	94.17%
82	P36	75.810898 M (98.776808%)	96.42%
83	P37	61.502986 M (98.304545%)	95.76%
84	P38	79.893598 M (98.784942%)	95.83%
85	P39	77.069784 M (98.669367%)	96.49%
86	P40	71.184256 M (98.680576%)	95.87%
87	P41	62.359794 M (98.821269%)	96.22%
88	P42	65.386370 M (98.900802%)	96.22%
89	P43	66.844432 M (98.557871%)	90.47%
90	P44	67.845070 M (98.808733%)	94.19%
91	P45	62.747668 M (98.648691%)	95.48%

92	P46	59.638662 M (98.173779%)	94.43%
**Mean Value**	**All Samples**	**69.00879 M (98.61430896%)**	**95.86%**

Table S4. Classification according to the breed of dogs that participated through the clinical efficacy evaluation of this study.

Species	General Feed [n (%)]	Diabetes Feed [n (%)]
Yorkshire terrier	0 (0)	2 (6.67)
Maltese	2 (12.50)	6 (20)
Shih-tzu	3 (18.75)	1 (3.33)
Spitz	1 (6.25)	3 (10)
Poodle	2 (12.50)	9 (30)
Chihuahua	4 (25)	1 (3.33)
Pomeranian	2 (12.50)	4 (13.33)
Bichon frise	0 (0)	2 (6.67)
Papillon	1 (6.25)	1 (3.33)
Dachshund	1 (6.25)	0 (0)
Mixed	0 (0)	1 (3.33)
Total	16	30

Table S5. Classification by gender and age of dogs through the clinical efficacy evaluation of this study.

Gender	General Feed [n (%)]	Diabetes Feed [n (%)]	Total [n (%)]
Unneutered female	6 (37.50)	4 (13.33)	10 (21.74)
Unneutered male	9 (56.25)	26 (86.67)	35 (76.09)
Neutered female	0 (0)	0 (0)	0 (0)
Neutered male	1 (6.25)	0 (0)	1 (2.17)
Age	7.44 ± 0.51	7.55 ± 0.50	7.51 ± 0.50

Mean ± standard deviation.

Table S6. Information of primer sequences in real time PCR of this study.

No.	Name		Sequences (5'- 3')	Size (bp)
1	ENSCAFG00000003029|CXCL8	F	CCCGTGTAGACCTGGTGTAG	234
		R	CCTGTAGGTGAGGTGGAAAGA	
2	ENSCAFG00000012970|MLXIPL	F	GTTCCCACTCTCTGCTCCTT	170
		R	ACAAAATCCACACACACGCA	
3	ENSCAFG00000009282|CREB3L1	F	TGAGTTCTCTGTCCAGTGCC	235
		R	TTCCACCGATGTCTTTTGGC	
4	ENSCAFG00000001254|EGR1	F	CGATCCTTTCTGCCCACTTG	187
		R	TCTAGCATCGAAGGGAGCAG	
5	ENSCAFG00000016107|NOTCH3	F	TCTGTGTATGGTGGCTGTGT	214
		R	CAGACCCGCAGAAATTCAGG	
6	ENSCAFG00000015708|ACTA2	F	TACTCTGTGTGGATCGGTGG	166
		R	ACAGAACGTTCACAATCGCG	
7	ENSCAFG00000013909|SERPINE1	F	CTGAGCACTGGGGAAGGTAA	169
		R	ACATGTGTGAGAGGGGTCAG	
8	ENSCAFG00000004008|IL2	F	GATGACGAGACAGCAACCAT	173
		R	ACAAAAGGTAGCAAAACGTACAT	

F is forward and R is reverse primer.

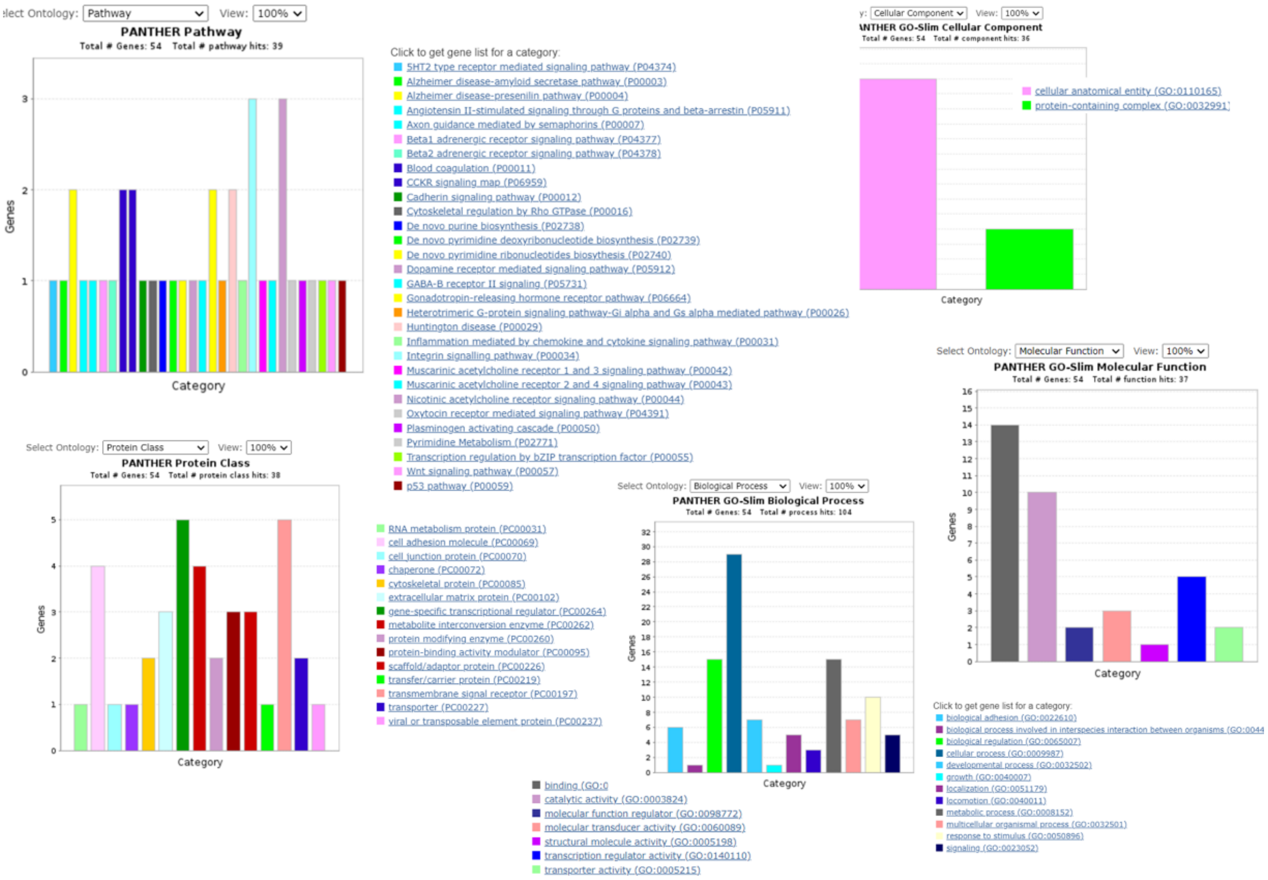

Figure S1: Functional enrichment of up regulated genes.

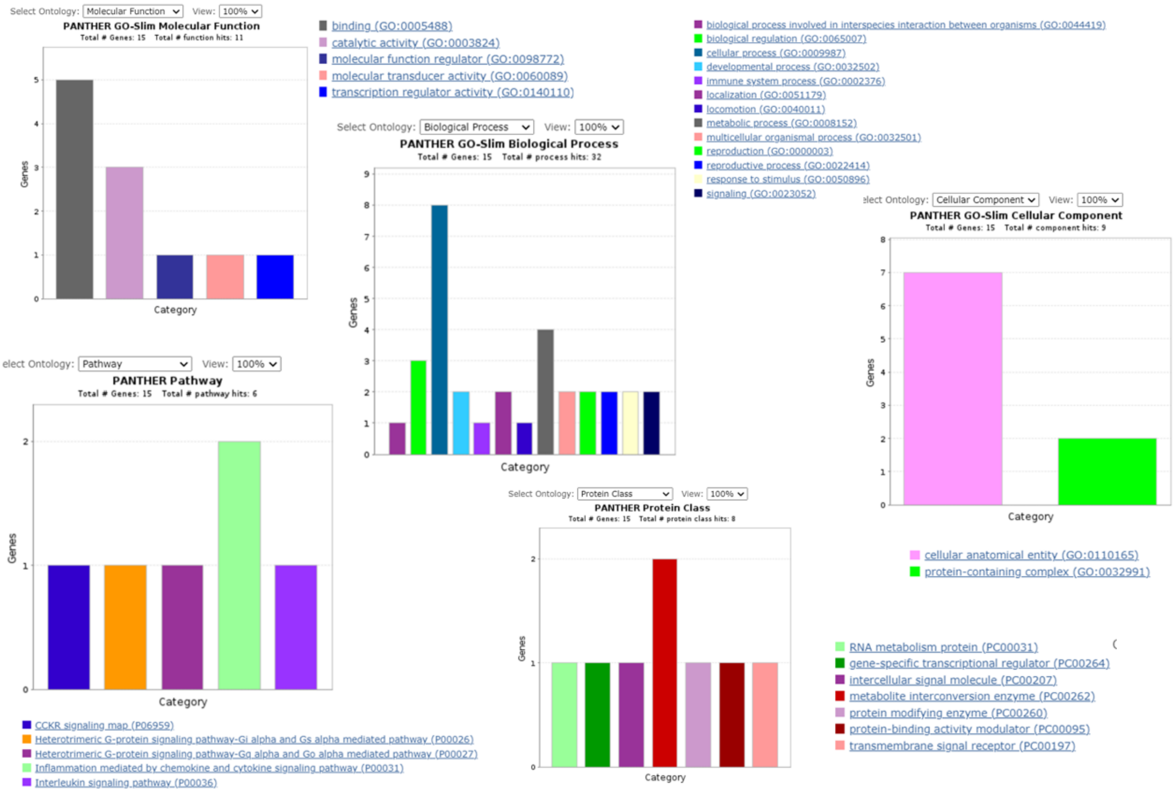

Figure S2: Functional enrichment of down regulated genes.

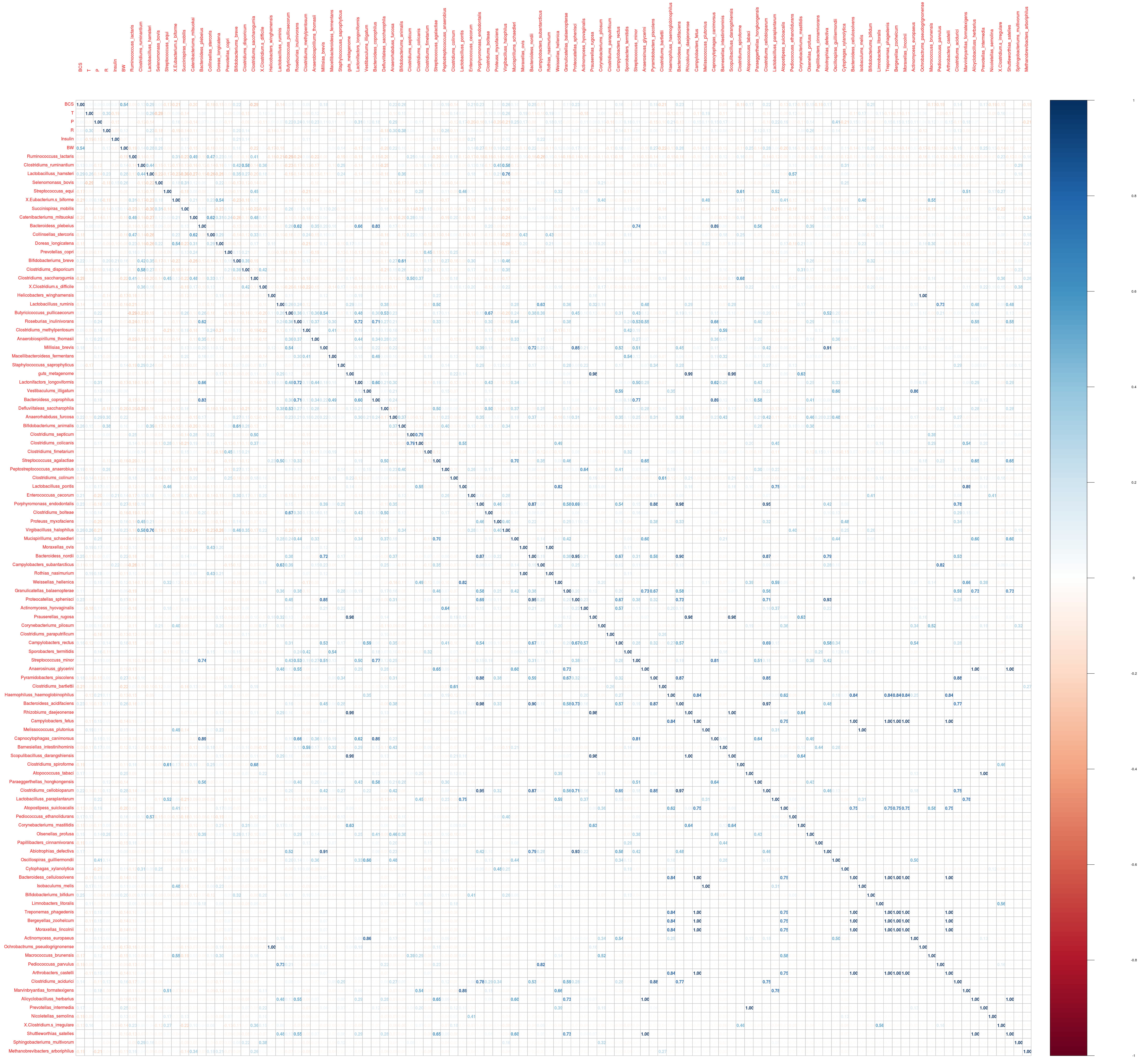

Figure S3. Visualization of correlation matrix among obesity-related factors and gut microbiome (species level)
